# Numerical Analysis of Zirconium and Titanium Implants under the Effect of Critical Masticatory Load

**DOI:** 10.3390/ma15217843

**Published:** 2022-11-07

**Authors:** Miguel Martinez-Mondragon, Guillermo Urriolagoitia-Sosa, Beatriz Romero-Ángeles, Daniel Maya-Anaya, Jacobo Martínez-Reyes, Francisco Javier Gallegos-Funes, Guillermo Manuel Urriolagoitia-Calderón

**Affiliations:** Biomechanics Group, Instituto Politecnico Nacional, Escuela Superior de Ingeniería Mecánica y Eléctrica, Sección de Estudios de Posgrado e Investigacion, Unidad Profesional Adolfo López Mateos. Edificio 5, 2do. Piso, Col. Lindavista, Del. Gustavo A. Madero, Ciudad de México 07300, Mexico

**Keywords:** dental implant, structural numerical analysis, biomodel development, tomography, abutment

## Abstract

Dental implants have become an alternative to replace the teeth of people suffering from edentulous and meet the physiological and morphological characteristics (recovering 95% of the chewing function). The evolution and innovation of biomaterials for dental implants have had a trajectory that dates back to prehistory, where dental pieces were replaced by ivory or seashells, to the present day, where they are replaced by metallic materials such as titanium or ceramics such as zirconium or fiberglass. The numerical evaluation focuses on comparing the stress distribution and general displacement between different dental implants and a healthy tooth when applying a force of 850 N. For the analysis, a model of the anatomical structure was developed of a healthy tooth considering three essential parts of the tooth (enamel, dentin, and pulp). The tooth biomodel was established through computed tomography. Three dental implant models were considered by changing the geometry of the abutment. A structural simulation was carried out by applying the finite element method (FEM). In addition, the material considered for the analyses was zirconium oxide (ZrO${}_{2}$), which was compared against titanium alloy (Ti${}_{6}$Al${}_{4}$V). The analyses were considered with linear, isotropic, and homogeneous properties. The variables included in the biomodeling were the modulus of elasticity, Poisson’s ratio, density, and elastic limit. The results obtained from the study indicated a significant difference in the biomechanical behavior of the von Mises forces and the displacement between the healthy tooth and the titanium and zirconium implant models. However, the difference between the titanium implant and the zirconium implant is minimal because one is more rigid, and the other is more tenacious.

## 1. Introduction

**Figure 1:**
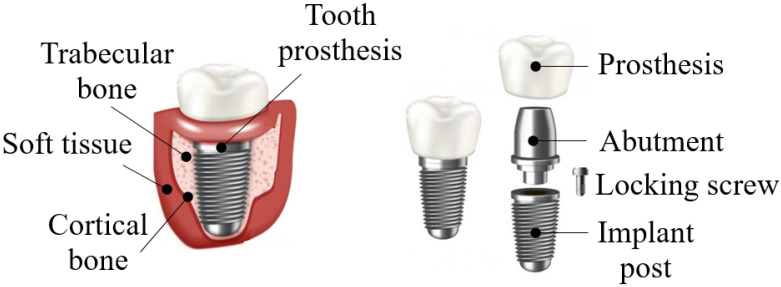
Dental implant with components.

In recent years, the growing demand for dental implant therapy has become increasingly popular due to its mechanical behavior and the potential to restore missing teeth without teeth grinding on the sides of the edentulous space [1]. In general, dental implants’ advantages are that they behave like natural teeth, last a lifetime, prevent bone loss, keep adjacent teeth stable, help keep you free of gum disease, and prevent facial sagging and premature aging. Nevertheless, the disadvantages of dental implants are the high cost, almost all dental insurance will not cover them, they require surgery for placement, and bone loss around the implants [2]. Concerning zirconia against titanium, the surface morphology is more important for osseointegration than the surface composition. To inhibit bacterial adhesion, zirconia is superior to titanium, and hence, more suitable for abutments. Both materials show similar capabilities for soft tissue adhesion, but zirconia tends to have less cost [3]. This has prompted research into new coatings techniques for better osseo-integration, modifying the surface of implants endowed with new physicochemical properties while also protecting against degradation, corrosion, friction, and tissue integration [4]. Excellent osseo-integration leads to a firm, direct, stable, and durable mechanical union between the bone and the body of a dental implant [5]. A dental implant is a component manufactured to optimize or replace the damaged or missing biological structure (dental piece) inserted within the bone tissue [6]. The dental implant consists of three elements: (1) implant, (2) bearing or abutment, and (3) prosthesis [7]. It has a screw-like appearance and a thread on the surface at the apex; in this manner, it is possible to increase the contact surface of the implant with the bone, as shown in Figure 1 [8,9]. In the dental area, biomaterials have an important role due to their technological advance and wide scientific research action field [10]. Currently, titanium and its alloys are the most widely used materials for prostheses manufactured for general use and teeth replacement due to their excellent biocompatibility, mechanical properties, and satisfactory results [11].

New technology development allows the elaboration of biocompatible materials, such as the case of ceramics, to meet the same characteristics that titanium alloys have [12]. However, ceramic materials tend not to be resistant to tensile and shear loads, promoting premature failure [13]. Currently, the material that covers these deficiencies is zirconium, which in recent years is an alternative suggested to be applied for dental implants [14]. Zirconium is a type of ceramic that developed in three crystallographic forms: monoclinic, tetragonal or metastable, and cubic [15]. In dentistry, the tetragonal form is applied because it has the best mechanical properties due to its crystalline structure, obtaining high toughness and resistance [16,17].

In this research, a numerical model of a dental piece was developed through computational tomography to be exported into a finite element method computer program for a structural evaluation. The numerical evaluation of the dental component will provide knowledge of the behavior close to reality with the interaction against chewing forces. Subsequently, three types of dental implants were proposed for evaluation, for which two types of materials were applied for each implant. The materials considered were titanium (Ti${}_{6}$Al${}_{4}$V) and zirconium (ZrO${}_{2}$) (both biocompatible materials). Finally, a result comparison between the cases of the study was performed, and conclusions regarding the implant and material with characteristics closer to the tooth are presented. The originality of this work is based on making a biomodel through computed tomography of the dental piece, evaluating it structurally, and comparing the service of various implants under the same conditions. Likewise, this methodology for developing bio models can be exported to diverse bone systems, being able to carry out structural evaluations and propose personalized rehabilitation methodologies.

## 2. Biomodeling Methodology

Biomodeling is a generic term that is understood as the ability to represent the morphological characteristics of an anatomical structure and biological systems in a physical model [18], and it has been a very important tool for the field of medicine and bioengineering. Biomodeling allows the visualization of bone structures to evaluate anatomy and biological functions by obtaining images with cross-sections of the human body [19]. Biomodels have been possible due to computed tomography (CT) contributions, which replaced conventional radiography [20].

**Figure 2:**
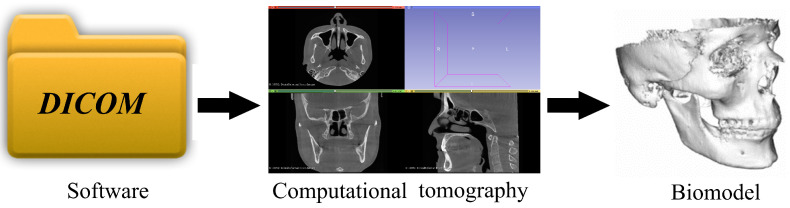
Development of biomodeling of anatomical structures.

In this research, it has been decided to develop a biomodel of a tooth to numerically simulate its behavior and compare it against dentary implant service. To create a three-dimensional model to be analyzed later, it has to go through several stages, and, as is known today, thanks to technology, countless computer programs allow everything from digital scanning to processing images in DICOM format to create volumetric modeling, as shown in Figure 2. All these computer programs have the necessary tools to develop biomodeling. However, most require knowledge to handle them, which would present a problem. As a result, a general methodology is presented that allows it to be applied to a case study for the bone system or anatomical structure, regardless of its difficulty [21].

**Figure 3:**
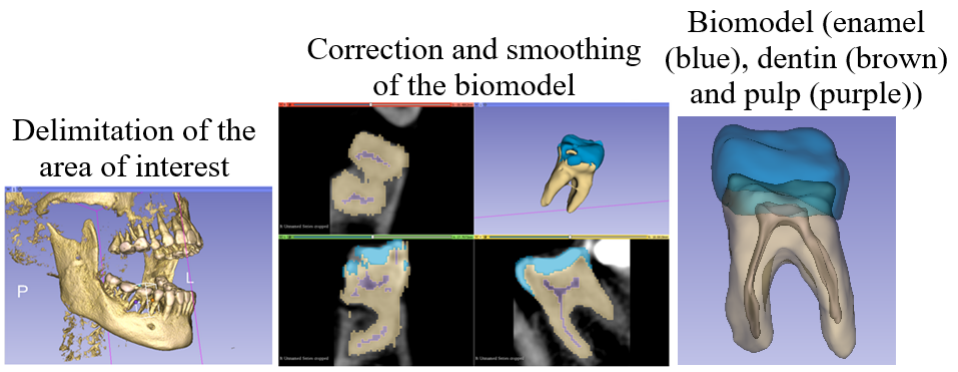
Modeling methodology.

The methodology is as follows [22]:

- Obtaining tomographic images of the anatomical structure to be analyzed.
- Importing images in DICOM format and modeling Figure 3.
- Importing the STL model into a CAD program (student version) to convert it to a solid.
- Assembly of solidified models.
- Export of CAD model to a Finite Element program for the development of the analysis.

**Figure 4:**
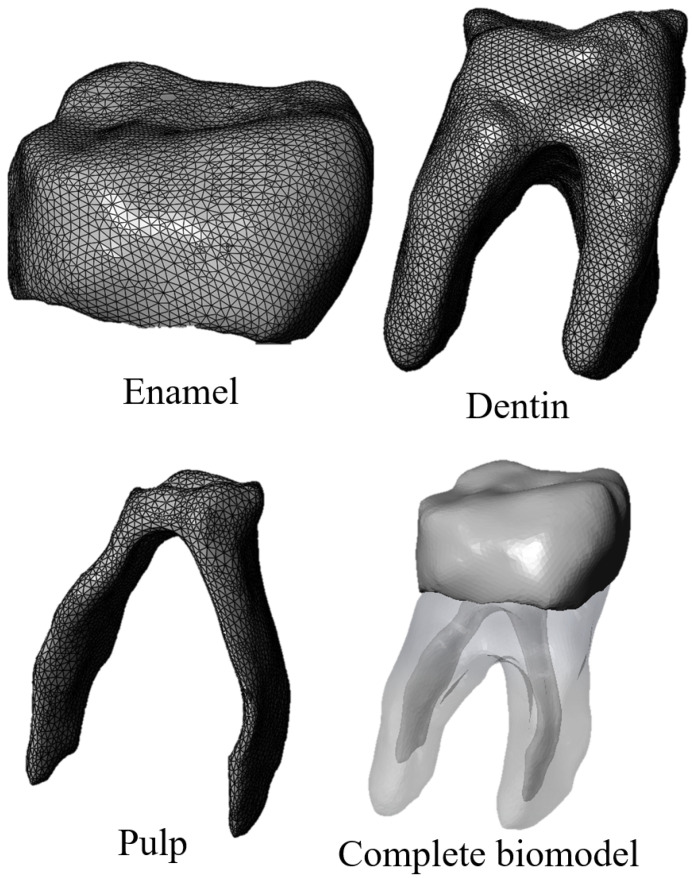
Biomodeling assembly.

After smoothing and exporting the biomodel, it is imported in STL format to a CAD program that allows it to solidify. Because a solid contains volume and thickness, allowing the addition of physical properties (density, weight, inertia, etc.) is something that is not possible with surfaces. The three solids (representing the tissues of the tooth) were assembled so that they could be presented as a single component, as shown in Figure 4 [23].

## 3. Materials and Methods

For this work, three simulations were carried out: the first one in a healthy tooth, the second in a titanium dental implant, and the third in a zirconium dental implant. The tooth biomodel was developed from a computational tomography of the first lower right molar of a 35-year-old female patient, apparently in a healthy state. It is important to state that this tooth was taken as a reference.

### 3.1. First Case of Study

**Figure 5:**
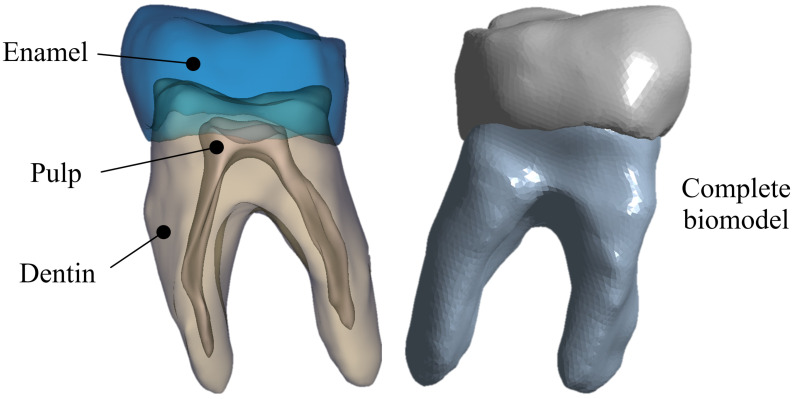
Biomodel of the lower right first molar.

**Figure 6:**
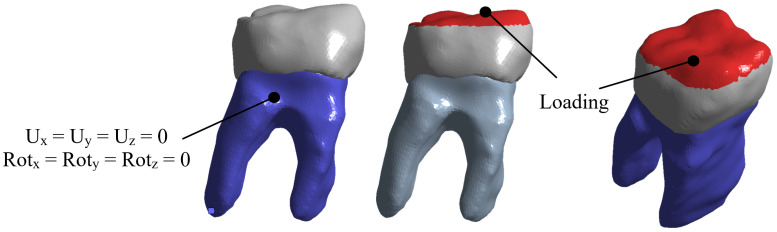
Boundary conditions and external loads on the molar.

For the first study case, an anatomical biomodel was developed Figure 5 from molar images obtained with 3D imaging using a cone beam computed tomography (CBTC) system. From the scan was obtained a digital volumetric tomography of the maxilla and mandible in a DICOM images file. This system is widely used in medicine and dentistry for the craniofacial region, allowing us to obtain tissue images that are difficult to observe [24]. Additionally, the study provides a better high-quality three-dimensional representation of the bone elements in the maxillofacial zone [25]. The model has high-order elements and is constructed with three different materials, corresponding to tissues that make up the dental organ (enamel, dentin, and pulp) [26]. Discretization of the biomodel was performed in a semi-automatic manner, with an element size of 0.2 mm${}^{2}$ and using tetrahedral elements throughout the model [27]. The mechanical properties of each bone tissue are described in Table 1 [28]. The bone tissues were considered structural materials with an isotropic characteristic and homogeneous internal structure. In addition, linear and elastic behavior was considered. Boundary conditions were applied in a constrained manner at the bottom of the tooth dentine area (Figure 6) according to the anatomical location of the molar roots, which are located within the alveolus in the mandibular bone.

For the application of the external agent, the chewing process was simulated, which is the contact that exists between the lower and upper molars, due to the movement of the jaw when compressing food. The load was applied in the contact area between the molars (in the occlusal face of the molar). The magnitude of the average chewing force of a person is 700 N. Nevertheless, a person suffering from bruxism can reach an 850 N load. For this case, it was considered the biggest load to produce a critical case of study. The load was distributed in the molar area in a form of pressure Figure 6.

### 3.2. Second and Third Case Studies

**Figure 7:**
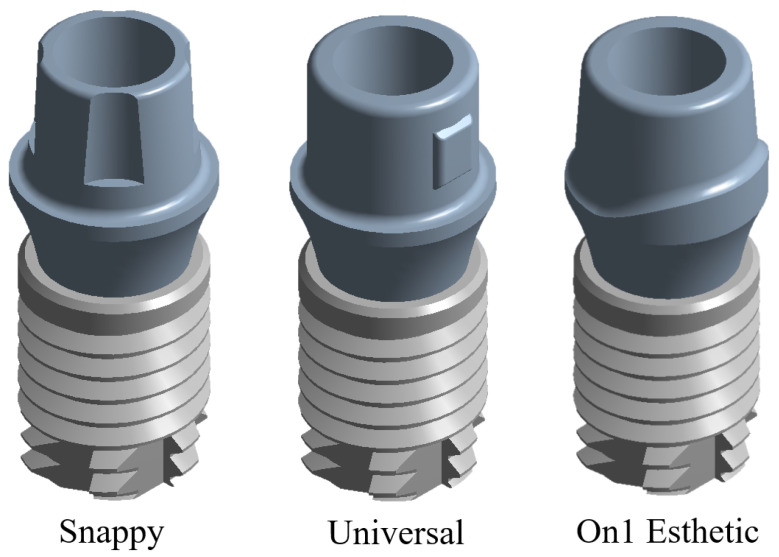
Implant models with different abutment geometry.

For the second and third case studies, three dental implants were developed by modifying the abutment geometry (Snappy, Universal, and On1 Esthetic), as shown in Figure 7. The tooth implant models mentioned above were produced based on a product developed by a commercial manufacturing company. The company which was chosen for the implants was Nobel Biocare located in Kloten, Switzerland (Swiss company), which is responsible for manufacturing dental implants and personalized prostheses [29]. As in the first case, the models have high-order elements and are built up of three pieces (implant, abutment, and union screw), which simulate the root of the molar. The discretization was carried out in the same manner as in the previous case (semi-automatically and with tetrahedral elements due to the geometry of the very acute angles). The mechanical properties are described in Table 2. It is worth mentioning that titanium (Ti${}_{6}$Al${}_{4}$V) was used for the second case and zirconium (ZrO${}_{2}$) was used for the third case. For the simulation of both cases, the isotropic and homogeneous material was considered with a linear–elastic behavior [30,31].

**Figure 8:**
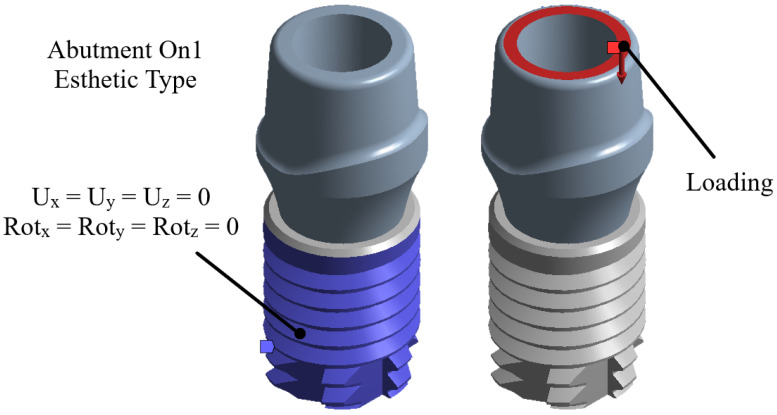
Boundary conditions and external loads on the implant.

Boundary conditions were applied to constrain the implant at the rear zone (Figure 8), which represents the osseo-integration between the bone and the implant. For the loading conditions, the same load of 850 N was applied to the area of the upper part of the pillar as pressure, as shown in Figure 8.

## 4. Results

The simulation of the force exerted by the bite on the occlusal area of the molar and the dental implant made it possible to analyze the total displacement and the Von Mises stress that occurs in the abutment area to visualize its behavior, obtaining the results shown in Table 3.

**Figure 9:**
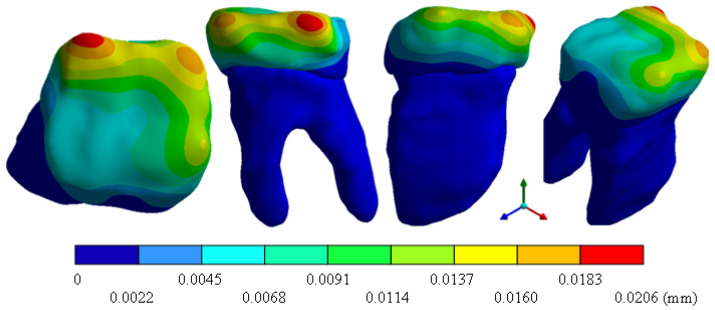
Total general displacement of the molar.

**Figure 10:**
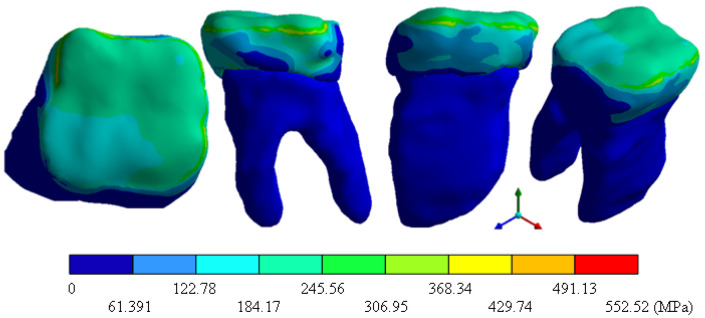
Von Mises stress for tooth.

**Figure 11:**
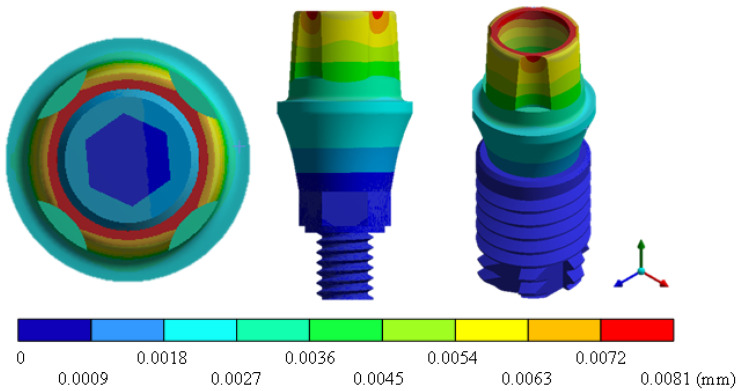
Total general displacement for Snappy type implant (Ti${}_{6}$Al${}_{4}$V).

**Figure 12:**
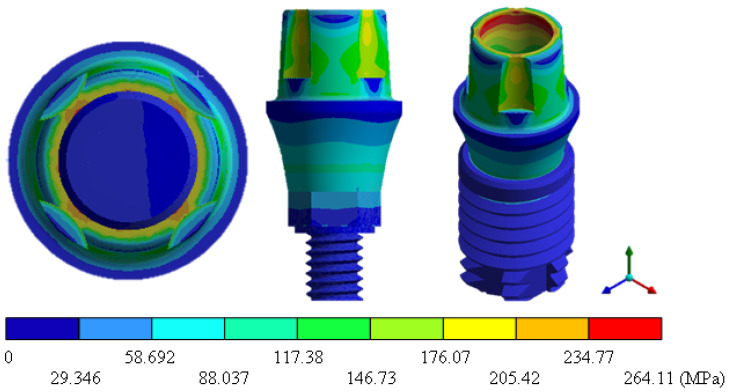
Von Mises stress for Snappy-type implant (Ti${}_{6}$Al${}_{4}$V).

**Figure 13:**
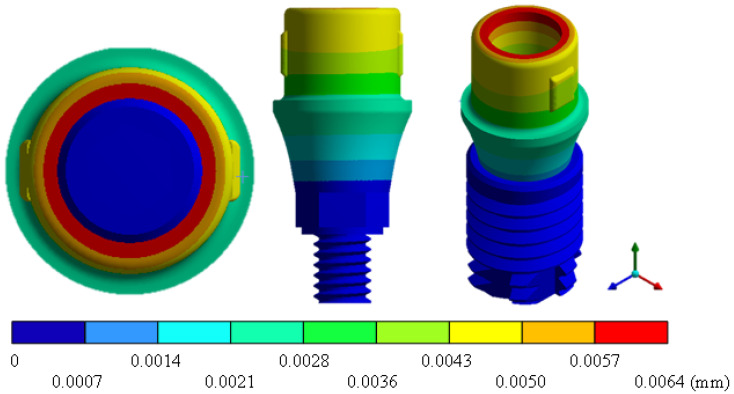
Total general displacement for Universal-type implant (Ti${}_{6}$Al${}_{4}$V).

**Figure 14:**
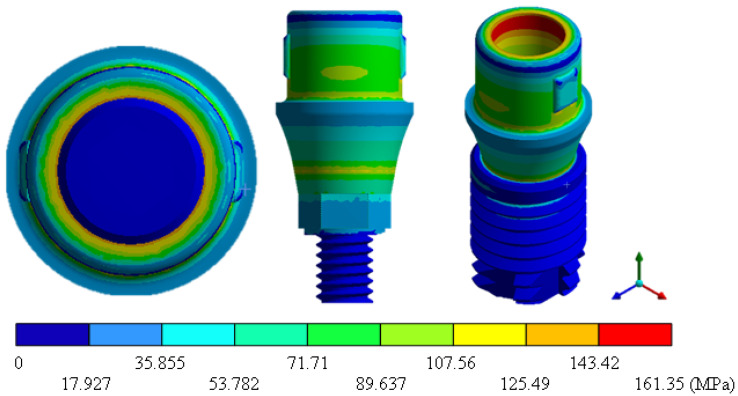
Von Mises stress for Universal-type implant (Ti${}_{6}$Al${}_{4}$V).

**Figure 15:**
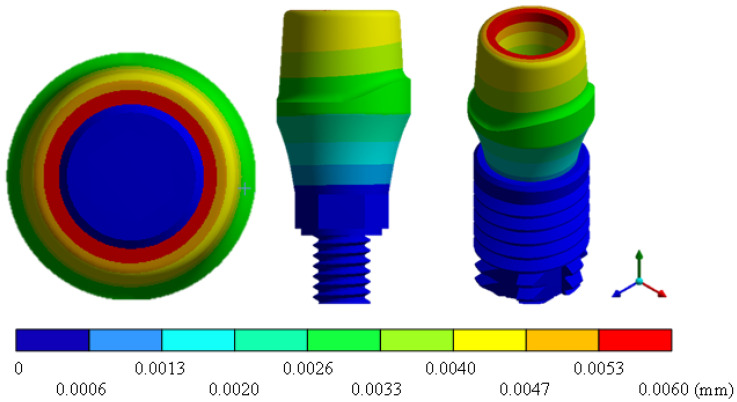
Total general displacement for On1 Esthethic-type implant (Ti${}_{6}$Al${}_{4}$V).

**Figure 16:**
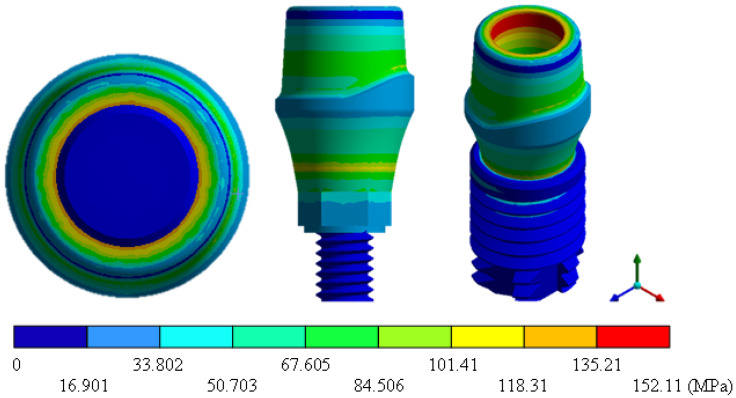
Von Mises stress for On1 Esthethic-type implant (Ti${}_{6}$Al${}_{4}$V).

**Figure 17:**
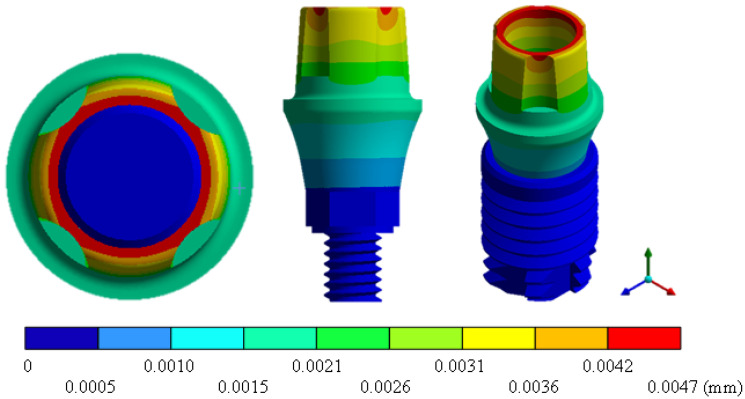
Total general displacement for Snappy-type implant (ZrO${}_{2}$).

**Figure 18:**
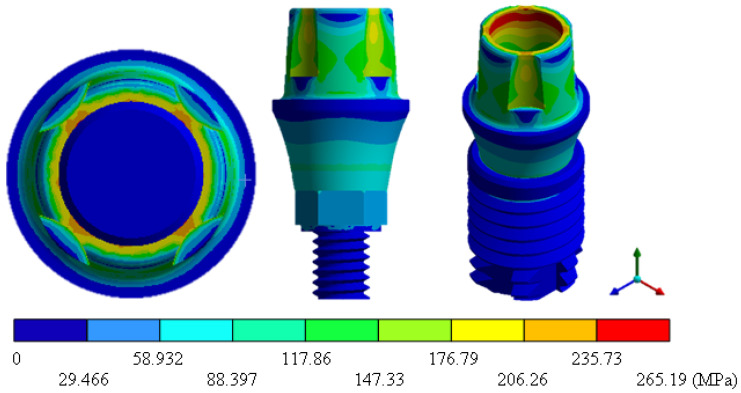
Von Mises stress for Snappy-type implant (ZrO${}_{2}$).

**Figure 19:**
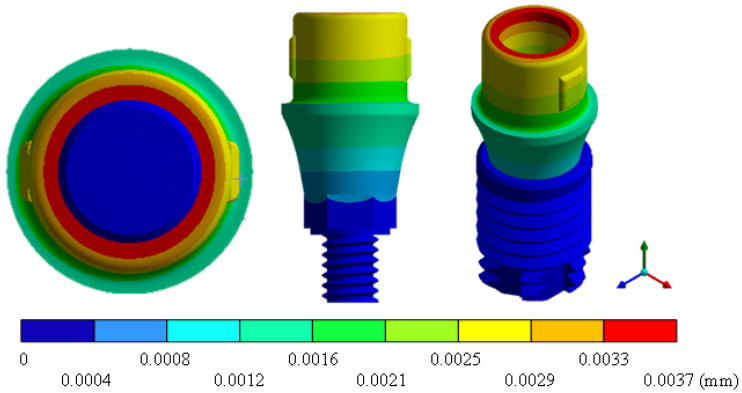
Total general displacement for Universal-type implant (ZrO${}_{2}$).

**Figure 20:**
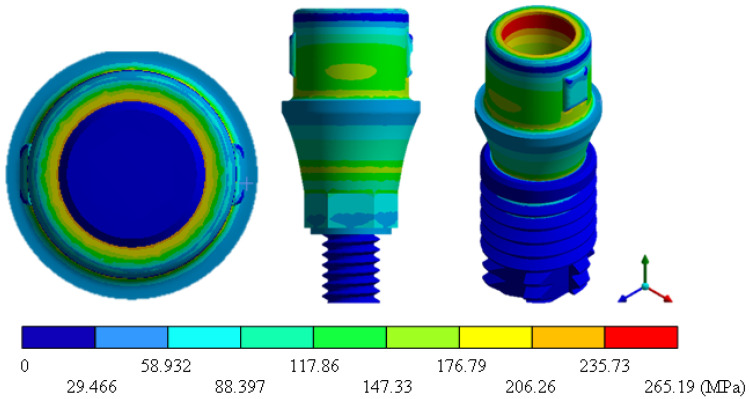
Von Mises stress for Universal-type implant (ZrO${}_{2}$).

**Figure 21:**
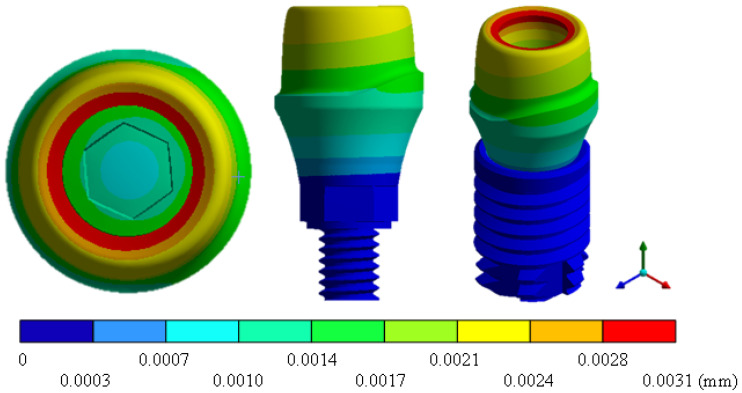
Total general displacement for On1 Esthetic-type implant (ZrO${}_{2}$).

**Figure 22:**
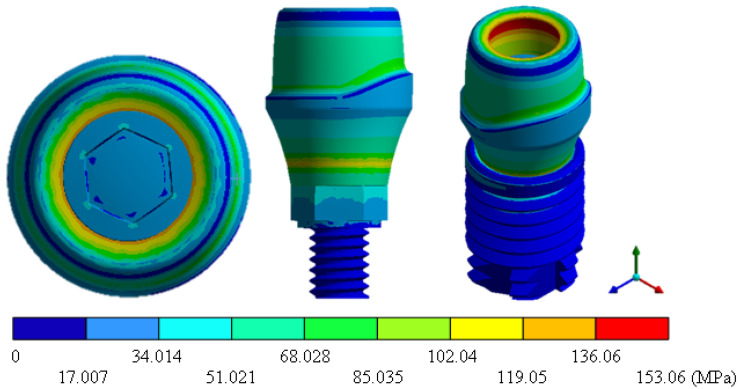
Von Mises stress for On1 Esthetic-type implant (ZrO${}_{2}$).

In the results of the simulation of the molar, Figure 9 shows the total general displacements that it had concerning the applied load, which indicates that it tends to move more through the lingual area, reaching a maximum of 0.02 mm. On the other hand, Figure 10 shows the area where the material will be more prone to failure and fracture. The maximum stress can be seen along the entire edge of the occlusal enamel area. In the case of the implant simulation (three different geometries and two different materials), it is considered that it is an ideal bite when the load acts vertically without having angular forces. So, in all implants, the mayor total general displacement occurs in the circumference of the upper part of the pillar. The magnitude of the total general displacement varies in each implant concerning material and geometry. By Von Mises stress, all implants present the same area where they will tend to fail and where it can be seen that the internal part is in tension and the external part is in compression For the results obtained from the simulation of the dental implant see Figure 11, Figure 12, Figure 13, Figure 14, Figure 15, Figure 16, Figure 17, Figure 18, Figure 19, Figure 20, Figure 21 and Figure 22.

## 5. Discussion

Currently, the area of mechanical engineering is applied globally and in almost all areas of study. However, there are areas where the depth of mechanical knowledge is not sufficient to provide satisfactory results. One of these areas is physiology, so the implementation of numerical biomodels allows the development of evaluations closer to reality and the possibility of implementing innovative procedures. The development presented in this work on a biomodel through computed tomography is innovative to be applied in all physical–mathematical sciences. For example, in the area of physiology, the implementation of numerical models would present a great diversity of development opportunities (cost reduction, procedure simplification, optimization of healing or rehabilitation methodology, evaluation of possible future failures, etc.). Likewise, depending on the degree of knowledge in mechanics, the models could be more complex and closer to reality. This research work is based on knowing the effects produced by the critical load that occurs in the chewing process (maximum load that is reached when biting). Therefore, a numerical structural evaluation is carried out between a healthy tooth and two dental implants (zirconium and titanium) with which it can be determined how close the behavior of the implants will be concerning the dental piece as well as, from the structural point of view, which of the implants can provide a better service. However, the components will have their advantages and disadvantages. The application of numerical evaluations through the finite element method will be able to quantify these and provide a database for researchers to make a better selection of components. This type of procedure can substantially reduce the experimental evaluation processes and all the regulations involved in experimentation with living beings (biological systems). In addition, the numerical procedure can be very user-friendly and simplify the knowledge base necessary to explain the effects that appear in the evaluation—the foregoing without counting the reduction in costs that this type of procedure involves. Finally, the authors consider that the methodology can be considered futuristic and that it has a great application for health problems when applied in conjunction with medicine, anatomy, biology, mechanics, physiology, etc. The authors have developed works applied to the rehabilitation and evaluation of bone failure in systems such as skull impact, knee failure, intervertebral disc tumors, rib blows, dental caries, gait evaluation, personalized endoprosthesis design, etc. (which can be seen in some of the references).

## 6. Conclusions

The cases of the study presented in this work have shown us the susceptible areas where the material can fail due to stress concentrators and stress intensifying. Despite this, the maximum stresses never exceeded the elastic limit under a single load condition. However, the mastication process has several load cycles and can fail due to fatigue. On the other hand, in the results presented, a change in stress and displacement was noted in each implant compared to the healthy tooth. Therefore, it allows us to question the importance and transcendence of biomaterials used in biomedical areas, and that they are so suitable to supply the functionality of any member of the human body. It is important to highlight the use of biomodels since they are specimens with a high morphology relationship with any member or organ of the human body. This is an efficient alternative for simulation tests before proceeding surgically.

In particular, the following conclusions were reached:

1. The model developed of the healthy tooth structure has 95% similarity to the morphology of a tooth. In conclusion, a good simulation and model of any organ, tissue, or structure of the human body can be obtained from a computational CT scan.
2. Ceramic implants are a great alternative for patients allergic to titanium; they prevent the formation of bacterial plaques and resist acid corrosion. In addition, their osseo-integration behavior and clinical survival rates are just as favorable as titanium implants.
3. For both materials, the physical and mechanical properties (titanium and zirconium) allow replacing the tooth structure, fulfilling its 100% functionality.
4. The general displacements of zirconium compared to titanium are less, because zirconium absorbs more impact energy and therefore is more tenacious.
5. The zirconium implant showed lower resistance to failure compared to the titanium one. However, the difference is not as significant and meets the objective of using ceramic materials instead of metallic ones.

The results obtained and the behavior observed in this presented study allows researchers to validate in a general way that through the application of the finite element method, it is possible to make simulations and analysis of complex models. Additionally, the analyses show that the use of technology has revolutionized more entirely when making decisions in the different biomedical areas. Appendix A Table A1 shows the complete results for this research.

## Figures and Tables

**Table 1:** Mechanical properties of the tooth.

Material	Young’s Modulus (MPa)	Poisson Ratio	Density (Kg/m^3^)
Enamel	70,000	0.30	250
Dentin	18,300	0.30	310
Pulp	2000	0.45	100

**Table 2:** Mechanical properties of titanium and zirconium.

Material	Young’s Modulus (MPa)	Poisson Ratio	Density (Kg/m^3^)	Elastic Limit (MPa)	Hardness (Hv)	Fracture Toughness (MPa m^1/2^)
Titanium	114	0.36	4430	1100	320	50
Zirconium	210	0.31	6100	900	1200	6–8

**Table 3:** Results obtained from the simulation in each case.

	Total General Displacement (mm)		Von Mises Stress (MPa)	
	Min	Max	Min	Max
Tooth (Molar)	0	0.0206	0	552.52
Implant Snappy (Ti${}_{6}$Al${}_{4}$V)	0	0.0081	0	264.11
Implant Universal (Ti${}_{6}$Al${}_{4}$V)	0	0.0064	0	161.35
Implant On1 Esthetic (Ti${}_{6}$Al${}_{4}$V)	0	0.0060	0	152.11
Implant Snappy (ZrO${}_{2}$)	0	0.0047	0	265.19
Implant Universal (ZrO${}_{2}$)	0	0.0037	0	162.01
Implant On1 Esthetic (ZrO${}_{2}$)	0	0.0032	0	157.65

## Data Availability

Not applicable.

## Abbreviations

The following abbreviations are used in this manuscript:

CAD	Computer-Aided Design
CBTC	Cone Beam Computed Tomography
CT	Computed Tomography
DICOM	Digital Imaging and Communication in Medicine
FEM	Finite Element Method
STL	STereoLithography

## Appendix A

**Table A1:** Complete results obtained from the simulation in general.

Value	Tooth	Ti${}_{6}$Al${}_{4}$V			ZrO${}_{2}$		
		Snappy	Universal	On1 Esthetic	Snappy	Universal	On1 Esthetic
Total strain (mm/mm)	0.0080 Max	0.0023 Max	0.0014 Max	0.0013 Max	0.0012 Max	0.0009 Max	0.0007 Max
	0 Min	0 Min	0 Min	0 Min	0 Min	0 Min	0 Min
Strain nominal in X (mm/mm)	0.0045 Max	0.0010 Max	0.0007 Max	0.0007 Max	0.0004 Max	0.0003 Max	0.0004 Max
	−0.0022 Min	−0.0003 Min	−0.0004 Min	−0.0004 Min	−0.0002 Min	−0.0002 Min	−0.0003 Min
Strain nominal in Y (mm/mm)	0.0019 Max	0.0004 Max	0.0002 Max	0.0002 Max	0.0002 Max	0.0002 Max	0.0002 Max
	−0.0072 Min	−0.0023 Min	−0.0014 Min	−0.0013 Min	−0.0012 Min	−0.0008 Min	−0.0007 Min
Strain nominal in Z (mm/mm)	0.0047 Max	0.0010 Max	0.0008 Max	0.0007 Max	0.0004 Max	0.0003 Max	0.0003 Max
	−0.0035 Min	−0.0003 Min	−0.0003 Min	−0.0004 Min	−0.0002 Min	−0.0001 Min	−0.0003 Min
Total displacement (mm)	0.0206 Max	0.0081 Max	0.0064 Max	0.0060 Max	0.0047 Max	0.0037 Max	0.0032 Max
	0 Min	0 Min	0 Min	0 Min	0 Min	0 Min	0 Min
Displacement nominal X (mm)	0.0064 Max	0.0012 Max	0.0008 Max	0.0006 Max	0.0007 Max	0.0004 Max	0.0003 Max
	−0.0025 Min	−0.0014 Min	−0.0009 Min	−0.0009 Min	−0.0008 Min	−0.0005 Min	−0.0006 Min
Displacement nominal Y (mm)	0.0002 Max	0.0002 Max	3.72 × 10${}^{-7}$ Max	3.90 × 10${}^{-7}$ Max	9.13 × 10${}^{-5}$ Max	3.86 × 10${}^{-7}$ Max	3.90 × 10${}^{-7}$ Max
	−0.0181 Min	−0.0081 Min	−0.0064 Min	−0.0060 Min	−0.0047 Min	−0.0037 Min	−0.0032 Min
Displacement nominal Z (mm)	0.0016 Max	0.0013 Max	0.0009 Max	0.0008 Max	0.0008 Max	0.0004 Max	0.0004 Max
	−0.0106 Min	−0.0013 Min	−0.0008 Min	−0.0007 Min	−0.0007 Min	−0.0004 Min	−0.0003 Min
Stress nominal X (MPa)	188.2 Max	61.56 Max	55.02 Max	57.67 Max	57.40 Max	51.1 Max	58.24 Max
	−294.1 Min	−139.8 Min	−107.6 Min	−105.5 Min	−137.2 Min	−102.9 Min	−103.1 Min
Stress nominal Y (MPa)	101.1 Max	54.79 Max	33.12 Max	27.75 Max	55.33 Max	30.47 Max	26.49 Max
	−617.1 Min	−282.9 Min	−184.2 Min	−174.7 Min	−280.8 Min	−183.7 Min	−202.8 Min
Stress nominal Z (MPa)	242.9 Max	61.62 Max	54.99 Max	56.49 Max	57.43 Max	50.40 Max	46.56 Max
	−468.7 Min	−136.4 Min	−103.2 Min	−107.7 Min	−133.4 Min	−98.39 Min	−104.7 Min
Maximum principal stress (MPa)	310.1 Max	63.81 Max	57.94 Max	58.02 Max	58.86 Max	55.64 Max	58.75 Max
	−291.2 Min	−75.78 Min	−58.21 Min	−52.13 Min	−75.85 Min	−57.96 Min	−54.95 Min
Minimum principal stress (MPa)	36.28 Max	11.67 Max	9.80 Max	11.94 Max	9.83 Max	8.56 Max	11.84 Max
	−710.7 Min	−282.9 Min	−198.8 Min	−174.8 Min	−280.8 Min	−194.9 Min	−204.8 Min
Shear stress XY (MPa)	126.8 Max	51.80 Max	61.25 Max	42.55 Max	52.75 Max	62.27 Max	39.88 Max
	−228.5 Min	−53.07 Min	−55.85 Min	−40.43 Min	−53.74 Min	−56.46 Min	−41.32 Min
Shear stress YZ (MPa)	225.5 Max	54.07 Max	46.70 Max	41.63 Max	55.11 Max	47.67 Max	40.67 Max
	−116.6 Min	−50.04 Min	−40.76 Min	−40.73 Min	−50.79 Min	−42.01 Min	−41.27 Min
Shear stress XZ (MPa)	114.4 Max	131.4 Max	51.80 Max	49.81 Max	127.5 Max	50.44 Max	49.28 Max
	−106.3 Min	−130.8 Min	−51.48 Min	−49.90 Min	−127.3 Min	−50.14 Min	−49.33 Min
Von Mises Stress (MPa)	552.5 Max	264.1 Max	161.3 Max	152.1 Max	265.1 Max	162.0 Max	157.6 Max
	0 Min	0 Min	0 Min	0 Min	0 Min	0 Min	0 Min
